# Identify Regioselective Residues of Ginsenoside Hydrolases by Graph-Based Active Learning from Molecular Dynamics

**DOI:** 10.3390/molecules29153614

**Published:** 2024-07-31

**Authors:** Yi Li, Hong-Qian Peng, Meng-Liang Wen, Li-Quan Yang

**Affiliations:** 1 College of Mathematics and Computer Science, Dali University, Dali 671000, China; liyigerry@163.com (Y.L.); phq1767045946@gmail.com (H.-Q.P.); 2 College of Agriculture and Biological Science, Dali University, Dali 671000, China; 3 School of Life Science, Yunnan University, Kunming 650091, China

**Keywords:** ginsenoside hydrolase, regioselectivity, active learning, graph neural networks, molecular dynamics

## Abstract

Identifying the catalytic regioselectivity of enzymes remains a challenge. Compared to experimental trial-and-error approaches, computational methods like molecular dynamics simulations provide valuable insights into enzyme characteristics. However, the massive data generated by these simulations hinder the extraction of knowledge about enzyme catalytic mechanisms without adequate modeling techniques. Here, we propose a computational framework utilizing graph-based active learning from molecular dynamics to identify the regioselectivity of ginsenoside hydrolases (GHs), which selectively catalyze C6 or C20 positions to obtain rare deglycosylated bioactive compounds from *Panax* plants. Experimental results reveal that the dynamic-aware graph model can excellently distinguish GH regioselectivity with accuracy as high as 96–98% even when different enzyme–substrate systems exhibit similar dynamic behaviors. The active learning strategy equips our model to work robustly while reducing the reliance on dynamic data, indicating its capacity to mine sufficient knowledge from short multi-replica simulations. Moreover, the model’s interpretability identified crucial residues and features associated with regioselectivity. Our findings contribute to the understanding of GH catalytic mechanisms and provide direct assistance for rational design to improve regioselectivity. We presented a general computational framework for modeling enzyme catalytic specificity from simulation data, paving the way for further integration of experimental and computational approaches in enzyme optimization and design.

## 1. Introduction

Plants of the genus *Panax*, belonging to the *Araliaceae* family, hold a prominent place in traditional Chinese herbal medicine due to their various pharmacological activities and therapeutic effects including anti-inflammatory, antioxidant, anticancer, neuroprotective, and immune regulation [1]. As the primary bioactive compound in the perennial plant *Panax*, ginsenosides have attracted widespread attention from both traditional medicine and modern pharmacology. More than 80% of naturally derived ginsenosides are glycosylated, involving numerous sites and types of glycosylation [2]. Clinical experiments have demonstrated that deglycosylated ginsenosides exhibit stronger pharmacological activities [3,4]. To promote molecular diversity and obtain rare deglycosylated bioactive compounds, many methods such as heating, acid hydrolysis, and enzymatic transformations have been developed to facilitate deglycosylation reactions.

**Figure 1:**
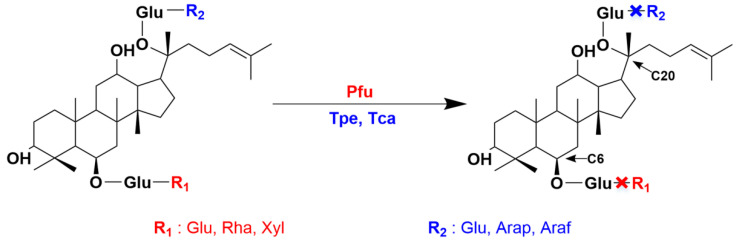
Regioselectivity of ginsenoside hydrolases. Ginsenoside hydrolases (Pfu, Tpe, Tca) with different regioselectivities can catalyze different sugar groups (R1 and R2) at the C6 and C20 positions of ginsenosides.

Considering its remarkable catalytic specificity, mild reaction conditions, and sustainable resources, enzymatic hydrolysis stands out as the most effective approach for achieving rare ginsenosides [5]. In this enzymatic conversion, glycoside hydrolases (GHs), an enzyme family responsible for selectively eliminating sugar groups at specific positions within ginsenoside molecules, play a central role (Figure 1). With the advancement of biochemical technology and metagenomic sequencing, a series of GHs have been successively discovered, remaining a research hotspot in the field of biological resources. As early as 2007, a β-glucosidase was unveiled from *Thermus Caldophilus,* named *Tcabgl1*. This enzyme can hydrolyze glucose, arabinopyranose, and arabinofurose glycosyl located outside the C20 position of ginsenosides [6]. In 2013, Oh et al. discovered another β-glucosidase, called *Pfubgl1*, isolated from *Pyrococcus Furiosus*, and characterized its capability to hydrolyze almost all common sugar groups at the C6 position of ginsenoside [7]. In 2019, a novel β-glucosidase (*Tpebgl1*) that can hydrolyze external glucose and arabinopyranose at the C20 position of ginsenosides was derived from *Thermotoga Petrophlia* [8].

Current research primarily focuses on discovering and characterizing GHs directly from natural living organisms. However, traditional enzyme discovery methods are haphazard, labor-intensive, and restricted by biological resources [9,10,11]. Naturally found GHs often fail to meet industrial requirements for the conversion of specific and rare ginsenosides [12]. In particular, these enzymes complicate production applications due to issues such as insufficient catalytic selectivity [13]. Furthermore, understanding the molecular basis behind the regioselectivity of GHs remains a significant challenge.

Compared to trial-and-error experimental methods, structure-based computational techniques, such as molecular docking, molecular dynamics (MD) simulations, and quantum mechanical calculations, can elucidate the regioselectivity of enzymes from the potential molecular mechanism and provide an in-depth understanding of structure–function relationships, enzyme–substrate interactions, and catalytic mechanisms. As the system size and time scale increase, MD simulations generate a large number of structural trajectories. For example, ligand dissociated from the receptor pocket and receptor conformation change need millisecond-long MD simulations that will generate a large amount of simulation data. However, it is difficult to use structure-based computational techniques to analyze such a large amount of data. Fortunately, machine learning (ML) can reduce the high-dimensional structure space to a low-dimensional feature space, improving the efficiency and speed of MD simulation data analysis [14]. For example, Plante et al. used ML methods to process a large amount of MD trajectory data to classify full agonist, partial agonist, and inverse agonist of GPCRs with high accuracy [15]. Ferraro et al. analyzed the MD ensemble through ML and successfully detected the dynamic patterns of allosteric inhibitor-bound and inhibitor-free TRAP1 [16]. Chuan Li et al. converted the MD trajectory into color images and used the convolutional neural networks to identify the different activity states of G proteins [17]. These studies demonstrate that ML models trained on simulated trajectories can achieve effective and accurate predictions at low cost.

In this study, we propose a graph-based active learning computational framework to identify regioselective residues of GHs from MD. Graph neural networks (GNNs) have been widely used for modeling relational data. The active learning strategy has been employed to systematically evaluate ML’s modeling ability on MD data. The key interaction residues between enzymes and substrates identified from MD trajectories are considered highly relevant to the regioselectivity of GHs, elucidating the molecular mechanisms underlying the broad substrate selectivity and regioselectivity of GHs. It is of great significance to develop regionally specific GHs for industrial applications. Our findings will contribute to a better understanding of the catalytic mechanisms of GHs and provide direct assistance for the rational design of GHs to enhance their regioselectivity. We demonstrate a general ML-driven computational framework for modeling enzyme catalytic specificity based on MD simulation data, paving the way for further coupling of experimental and computational approaches in enzyme optimization and design.

## 2. Materials and Methods

### 2.1. Experimental Data Collection and Structure Acquisition

To preserve consistency in the genetic background, GHs with distinct regioselectivity while belonging to the same structural architecture, i.e., the (β/α)_8_-barrel structural architecture in glycoside hydrolase family 1, were screened based on literature reports [6,7,8]. To balance the regioselectivity and glycosyl type, three GHs were finally selected: *Pfubgl1* (Pfu), *Tpebgl1* (Tpe), and *Tcabgl1* (Tca). Among these GHs, Pfu (GenBank ID: AAC25555.1) stands out for its specificity in catalyzing the C6 outer sugar group, exhibiting insensitivity to various glycan types such as glucose (glu), xylose (xyl), and rhamnose (rha). There are two GHs, Tpe and Tca, both targeting sugar groups located outside C20. The GenBank IDs of Tpe and Tca are ABQ46970.1 and AAO15361.1, respectively. Tpe hydrolyzes glu and arabinopyranose (arap) sugar groups, whereas Tca demonstrates catalytic activity towards three type sugar groups, i.e., glu, arap, and arabinofurose (araf).

The amino acid sequences (Supplementary Figure S1) of Pfu, Tpe, and Tca were obtained from the UniProt database [18] with IDs Q51723, A5IL97, and Q8GHE5, respectively. Based on the catalytic annotation provided by the UniProt, the catalytic residues of Tpe are E166 and E351, while those residues in Tca consist of E164 and E338. By structurally aligning with Tpe and Tca, the catalytic residues of Pfu were inferred as E207 and E372. Three-dimensional structures of Pfu, Tpe, and Tca predicted by the AlphaFold2 were accessed from the AlphaFold Protein Structure Database [19].

Structures of glycosylated ginsenosides were obtained from the PubChem database [20], with accession numbers as follows: R1 (80418-24-2), R2 (80418-25-3), Re (52286-59-6), Rf (52286-58-5), Rg2 (52286-74-5), Rb1 (41753-43-9), Rb2 (11021-13-9), and Rc (11021-14-0). The RDKit software (version 2023.3.2) package (https://www.rdkit.org, accessed on 1 February 2024) was employed to handle molecular structures. The structures of all substrate molecules are shown in Supplementary Figure S2.

Sequences of Pfu, Tpe, and Tca were aligned using the MAFFT online tool [21], and the results were visualized using ESPript (version 3.0) [22]. For structural superimposition and display, Pymol (version 2.5.4) software (https://pymol.org, accessed on 1 February 2024) was employed.

### 2.2. Molecular Docking and Validation

Complexed structures of ginsenoside hydrolase and its corresponding glycosylated ginsenoside were constructed through molecular docking and validation. The docking process was implemented using AutoDock Vina 1.1.2 [23]. The grid center coordinates of a cubic simulation box were placed at the geometric center of the catalytic residues for each enzyme. The box size of 40 × 40 × 40 Å was designed to encompass all substrates with various glycosyl types. Proteins and ligands were prepared using prepare_receptor4.py and prepare_ligand4.py in MGLtools (Version 1.5.6) (https://ccsb.scripps.edu/mgltools, accessed on 1 February 2024), respectively. For each enzyme–substrate pair, 5000 docking poses were generated employing an exhaustiveness of 25, an energy_range of 10, and a num_modes of 20, while other parameters remained at their default values. Following the docking procedure, docking results were meticulously analyzed and selected regarding the crystal structure of the glycoside hydrolase family.

### 2.3. Molecular Dynamics Simulations

All MD simulations were performed by GPU-accelerated programs in Amber 22 [24]. Structural refinements of the enzyme and parameterizations of the substrate were accomplished by ‘pdb4amber’ and ‘reduce’ commands in Amber tools, respectively. Each system was individually solvated using TIP3P water molecules [25] in an octahedral box, ensuring a minimum distance of 10 Å between the box wall and any solute atom. The ‘addIons2’ command in ‘tleap’ automatically adds the corresponding number of Na^+^ or Cl^−^ atoms to make the system in a charge-balanced state. The solvated system was minimized using 500 steps of steepest descent followed by 1500 steps of conjugated gradient methods. The bonded and nonbonded parameters of the enzyme and substrate were treated with the AMBER ff14SB force field [26] and the generalized AMBER force field version 2 [27], respectively.

For each enzyme–substrate system, three replicas (r1–r3) of 100 ns production MD simulations were carried out following a standardized protocol. Atomic velocities generated by the Maxwell distribution at 310 K were initialized for each replica. The SHAKE algorithm was applied to constrain all covalent bonds involving hydrogen atoms [28]. The van der Waals and short-range electrostatics were cut off at 12.0 Å with a switch at 10.0 Å. The particle mesh Ewald summation method was employed for long-range electrostatic interaction [29]. Protein and non-protein components were independently coupled to a 310 K and 1 atm with an external bath. The whole system was simulated under the isothermal–isobaric ensemble with a time step of 2 fs until reaching 100 ns. The system coordinates were saved as a snapshot every 10 ps for the following analysis. All simulation systems are shown in Table 1.

### 2.4. Trajectory Analysis Methods

The trajectory was analyzed by Amber’s tool ‘cpptraj’ and the Prody package [30]. All results were presented using custom Python scripts based on the matplotlib [31] and seaborn (https://seaborn.pydata.org, accessed on 1 February 2024) libraries. The solvent-accessible surface area (SASA) was computed using cpptraj’s ‘surf’ command. RMSD (root-mean-square deviation) and RMSF (root-mean-square fluctuation) values were conducted utilizing the ‘rmsd’ and ‘atomicfluct’ commands in ‘cpptraj’, respectively. Before RMSD and RMSF calculation, all trajectories were aligned to their respective initial structure to reduce the impact of overall translation and rotation. Only Cα atoms were involved in computing RMSD and RMSF.

The formula of RMSD is:

$$\mathrm{RMSD}=\sqrt{\frac{1}{N}\sum_{i=1}^{N}{\left(x_{i}-y_{i}\right)}^{2}}$$

where N is the number of Cα atoms, and $x_{i}$ and $y_{i}$ denote the coordinate vectors of the i th Cα atom in the snapshot and initial structure, respectively.

For the i th Cα atom, its RMSF is calculated by:

$${\mathrm{RMSF}}_{i}=\sqrt{\frac{1}{T}\sum_{t=0}^{T}{\left(x_{i}^{t}-\langle x_{i}\rangle \right)}^{2}}$$

where T is the total number of snapshots in a trajectory, $x_{i}$ denote the position vectors of the i th Cα atom in the snapshot t, and $\langle x_{i}\rangle$ is the average position vector of the i th Cα atom over all snapshots.

The ‘cluster’ command in ‘cpptraj’ was used for clustering, with the algorithm set to DBSCAN algorithm, min points set to 25, and epsilon set to 0.9. The binding modes of the representative structures were analyzed using LigPlot software (version 2.2.8) [32].

### 2.5. Graph Neural Network

A heterogeneous graph involving two types of nodes, substrate and enzyme residue, was employed to model the molecule interaction between ginsenoside hydrolase and glycosylated substrates. The entire substrate molecule was abstracted as a substrate node. All residues whose Ca atom is located less than 5 Å away from any atom of the substrate in the initial structure were modeled as residue nodes. In this heterogeneous graph, there are two types of edges to describe interactions between the substrate and enzyme residues and interactions among enzyme residues. For the first type of edge, the substrate node was connected to each residue node. Residues that interact with each other were connected by edges between residue nodes based on a typical distance between their Ca atom pairs smaller than 7 Å.

Each snapshot in every trajectory was modeled as a heterogeneous graph. Substrate node was characterized by the one-hot encoding based on regioselectivity and glycan type. Residue node features include covalent interactions (bond energy, angle, dihedral angle) and non-covalent interactions (van der Waals, electrostatic interactions), which were calculated by the energy command in ‘cpptraj’.

The learning process was implemented using the PyTorch Geometric [33] library and the PyTorch [34] framework. A GNN model comprising three SageConv layers [35], one dropout layer, and one linear layer was constructed to classify the regioselectivity, i.e., C6 or C20, of GHs. The loss function adopted the cross-entropy. The optimization for learning utilized Adam. To fine-tune the learning rate effectively, 40 rounds of Bayesian optimization followed by 10 rounds of random search were employed to iteratively refine it within the hyperparameter space ranging from 0.01 to 0.0001.

In the GNN model, the message-passing process in the l-th layer is shown in the following:

$$h_{v}^{l}\;=\begin{array}{c} ⊕\\ r\in R \end{array}f_{\theta }^{\left(l,r\right)}\left(h_{v}^{l-1},\left\{h_{w}^{l-1}:w\in N^{r}\left(v\right)\right\}\right)$$

where $f_{\theta }^{\left(l,r\right)}$ represents the relation instance, $N^{r}\left(v\right)$ represents all neighboring nodes of vertex v under relation r, and ⊕ represents the aggregation function.

The first-replica trajectories of Pfu complexed with R2, Re, or Rf, Tpe complexed with Rb2, and Tca complexed with Rb1 or Rc were selected as the training set. The GNN model was trained by 5-fold cross-validation. The trajectories of the second and third replicas were used as the replica test set. The first-replica trajectories of Pfu complexed with R1 and Rg2, Tpe complexed with Rb1, and Tca complexed with Rb2 are selected as the glycan test set.

### 2.6. Uncertainty Calculation

The epistemic uncertainty was used to assess the uncertainty caused by the fact that the parameters and structure of the model may not be completely accurate [36]. Using Monte Carlo dropout, the model generates slightly different predictions for the same sample over multiple iterations. The model’s uncertainty is determined by calculating the variance of these predictions.

### 2.7. Evaluation Metrics

To evaluate the classification performance of GHs’ regioselectivity, a confusion matrix comprising four elements, including true positive (TP), false positive (FP), false negative (FN), and true negative (TN), was used. TP indicates the number of samples correctly identified as positive by the classifier, while FP indicates the number of negative samples mistakenly classified as positive. FN represents positive samples incorrectly classified as negative, and TN denotes correctly identified negative samples. Based on the confusion matrix, four evaluation metrics, including precision, recall, accuracy, and Matthews correlation coefficient (MCC), were calculated by:

$$\mathrm{Precision}=\frac{TP}{TP+FP}$$

$$\mathrm{Recall}=\frac{TP}{TP+FN}$$

$$\mathrm{Accuracy}=\frac{TN+TP}{TN+TP+FN+FP}$$

$$\mathrm{MCC}=\frac{TP*TN-FP*FN}{\sqrt{\left(TP+FP\right)\left(TP+FN\right)\left(TN+FP\right)\left(TN+FN\right)}}$$

### 2.8. Interpretability Algorithm

A mask strategy was used to filter out the subgraphs that have the greatest impact on model performance [37]. The Captum algorithm that supports heterogeneous graphs was employed to extract model interpretability [38]. The fidelity score was selected as an evaluation indicator for the reliability of interpretability. The subgraph’s contribution to the prediction was evaluated by comparing fidelity scores before (fid_+_) and after (fid_−_) removing the important subgraph. Here, fid_+_ represents necessity. If removing the subgraph from the initial graph changes the model’s prediction, it also indicates the subgraph’s importance. Fid- represents sufficiency. If the presence of a subgraph alone leads to the model’s initial prediction, it indicates the subgraph’s importance. The specific formulas are as follows:

$$fid_{+}\;=\frac{1}{N}\sum_{i=1}^{N}1\left(\hat{y_{i}}=y_{i}\right)-1\left({\hat{y_{i}}}^{G_{C\backslash S}}=y_{i}\right)$$

$$fid_{-}\;=\frac{1}{N}\sum_{i=1}^{N}1\left(\hat{y_{i}}=y_{i}\right)-1\left({\hat{y_{i}}}^{G_{C}}=y_{i}\right)$$

where $y_{i}$ and $\hat{y_{i}}$ are the label and prediction of graph i, respectively. N is the number of graph samples. ${\hat{y_{i}}}^{G_{C}}$ and ${\hat{y_{i}}}^{G_{C\backslash S}}$ represent the prediction of the model when the subgraph is retained and removed, respectively. When $\hat{y_{i}}$ and $y_{i}$ are equal, 1 is returned, otherwise, 0 is returned.

## 3. Results and Discussion

### 3.1. Sequence and Structure Alignments of Ginsenoside Hydrolase

**Figure 2:**
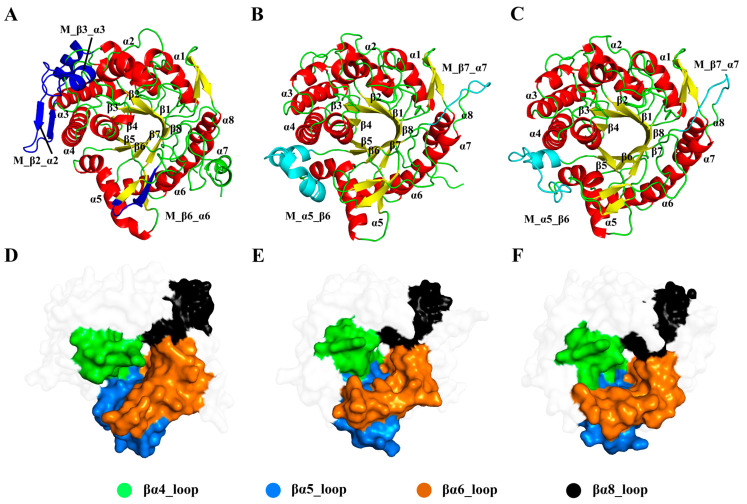
Comparison of the structures of ginsenoside hydrolases. Cartoon representations of the Pfu (**A**), Tpe (**B**), and Tca (**C**) with missing parts in blue and cyan. Surface representations of Pfu (**D**), Tpe (**E**), and Tca (**F**) with the loop regions on the active site in different colors.

GHs with different regioselectivities share a typical (β/α)_8_-barrel structural architecture, which is commonly found in glycoside hydrolase family 1 (Figure 2). This structural form, known as the TIM-barrel fold, was first discovered in the triosephosphate isomerase (TIM) [39]. Despite the high similarities in their core structures, the selected GHs exhibit several insertions or deletions in the peripheral rings, labeled as M regions (Figure 2A–C). In Pfu, fragments of the sequence were inserted into the βα2, βα3, and βα6, denoted as M_β2_α2, M_β3_α3, and M_β6_α6, respectively. For Tpe and Tca, insertions occur between α5 and β6, and within βα7, recorded as M_α5_β6 and M_β7_α7, respectively. These insertions or deletions in peripheral loop regions will influence the structural flexibility of GHs.

In the (β/α)_8_-barrel structure, the active site contains two conserved carboxylic acid residues on β strands 4 and 6, serving as the catalytic acid/base and nucleophile, respectively. Additionally, the elongated molecular structure of ginsenosides is associated with the loop regions around the catalytic surface, particularly concentrated in the loops in βα5 and βα8. Structural analysis showed that βα4, βα5, βα6, and βα8 (highlighted in Figure 2D–F) are closely related to the substrate specificity of GHs.

### 3.2. Cluster Analysis of Substrate Docking Poses

**Figure 3:**
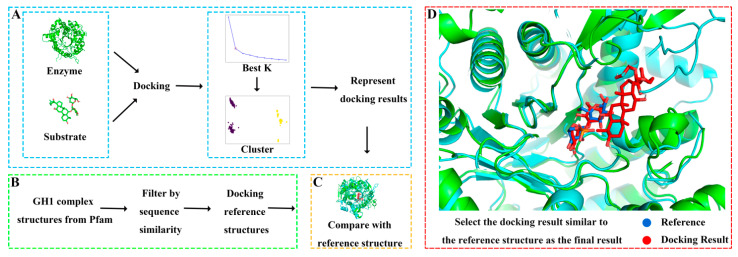
Molecular docking process. (**A**) Obtaining representative docking poses by clustering a large number of docking results. (**B**) Screening reference structures from glycoside hydrolase family 1. (**C**) Selecting the docking pose closest to the reference structure as the final result. (**D**) Local comparison of the final docking result of ginsenoside hydrolase Pfu and substrate R1 with the reference structure 8B81.

Due to the lack of experimentally resolved substrate-complexed GH structures, experiment-resolved structures of glycoside hydrolase family 1 with substrates were selected as references to validate the docking results. The steps for selecting reference experimental structures were as follows (Figure 3): (1) Obtaining all experimental structures of glycoside hydrolase family 1. (2) Identifying experimental structures with polysaccharide substrates. (3) Since ginsenosides have two sugar rings, experimental structures with substrates containing two sugar rings were further screened. (4) Excluding structures with high sequence similarity or those that are too long or too short. (5) Ensuring that the catalytic residues are as similar as possible to those of GHs. Ultimately, three reference experimental structures complexed with substrates were obtained, with PDB IDs of 2O9R, 4PTV, and 8B81, respectively.

Different glycosylated ginsenoside substrates were docked to GHs. After aligning with the reference structure, the distance between every docking result and the glycosidic bonds in the reference structure was calculated. All docking results within 5 Å of the glycosidic bond distance to the reference were screened out, and then these docking results were clustered. The glycoside hydrolases operate substrates by two acidic residues: one as a general acid residue and the other as a nucleophile. The spatial position between the glycosidic bond in the substrate and the active residues of the enzyme is a critical factor affecting catalysis [40]. Therefore, we validated and selected the docking results based on the distance between the glycosidic bonds between the docking results and the reference structure. The docking result with the shortest distance in each cluster is chosen as the representative structure. By comparing the orientation of each representative structure and the reference structure, the structure most similar to the reference structure was selected as the final docking result. For example, the final docking result of Pfu and R1 was compared with the reference structure 8B81 with the docking energy of −8.4 kcal/mol and the distance between the glycosidic bond of 0.65 Å, yielding highly reliable docking results. Detailed docking results of enzymes and substrates are shown in Supplementary Table S1 and Figure S3.

### 3.3. Different Systems Exhibit Similar Dynamic Macroscopic Properties

**Figure 4:**
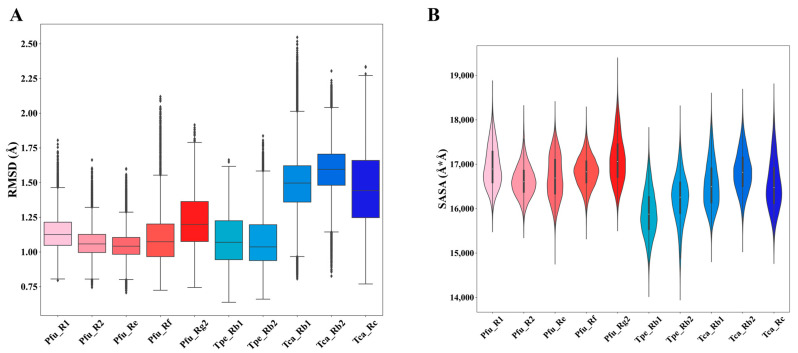
Statistical analysis of dynamic properties in molecular dynamics simulations of the ginsenoside hydrolases (Pfu, Tpe, and Tca) complexed with different substrates (R1, R2, Re, Rf, Rg2, Rb1, Rb2, Rc). (**A**) Root-mean-square deviation (RMSD). (**B**) Solvent-accessible surface area (SASA).

To evaluate the molecular dynamic differences of GHs with different regioselectivities, we statistically analyzed the macroscopic dynamic properties represented by RMSD and SASA in each simulation system (Figure 4). RMSD is a quantitative indicator to compare the structural differences between proteins during simulations and reference structures, assessing simulation stability and convergence. RMSD values of all simulation systems are between 0.75 and 1.75 Å with minor fluctuations, indicating that the simulation systems are very stable (Figure 4A). Among them, Pfu and Tpe have similar RMSD distributions, ranging from 1.0 to 1.5 Å. The RMSD value of Tca (1.25–1.75 Å) is slightly higher than those for Pfu and Tpe. The RMSD comparison indicates that there is no significant difference between GHs with different regioselectivities when complexed with different substrates.

SASA is a commonly used statistical measure to characterize the exposed surface area of proteins. Based on SASA, the solubility, metal ion coordination ability, surface activity, and other properties of proteins can be evaluated. As shown in Figure 4B, the SASA of Pfu and Tca is distributed between 16,000 and 18,000 ${Å}^{2}$, while the SASA of Tpe is distributed between 15,000 and 17,000 ${Å}^{2}$, indicating that there is no obvious difference among the three in terms of SASA properties. Combined with the comparison of RMSD, it is clear that GHs with different regioselectivities exhibit similar macroscopic dynamic properties.

### 3.4. Residue Dynamics Exacerbate the Challenge of Distinguishing Regioselectivity

**Figure 5:**
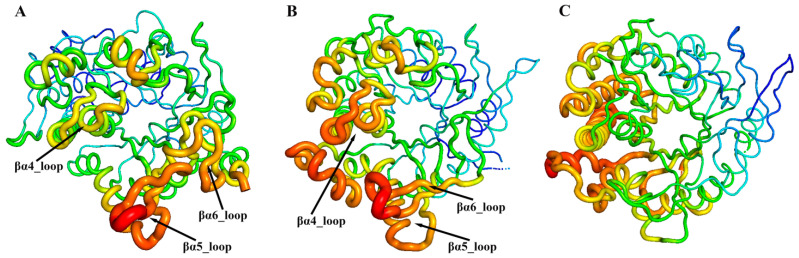
Conformational dynamics of ginsenoside hydrolases, Pfu (**A**), Tpe (**B**), and Tca (**C**). 3D backbone representation structures mapped with per-residue average backbone RMSF (root-mean-square fluctuation) values. The backbone color ranges from red to blue corresponding to line thickness, and denotes backbone RMSF values varying from lowest to highest.

By comparing and analyzing RMSF during the MD simulations, the flexibility and dynamics of different regions in the enzyme can be revealed, providing crucial insights into understanding the relationship between molecular structure and function. As shown in Figure 5, the regions with higher flexibility are located in the βα4_loop, βα5_loop, and βα6_loop around the active site in the Pfu and Tpe. However, in the Tca, the areas with greater flexibility are concentrated in the outer α-helical regions near the βα4, away from the active site. The clustering of highly flexible regions near the active site suggests that these loop regions have some special relationship with the substrate specificity of GHs.

### 3.5. Classification Performance of Graph Neural Network

**Figure 6:**
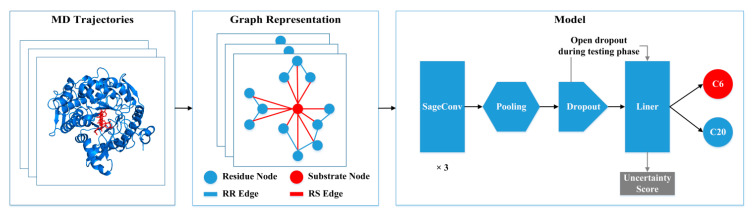
The overall architecture of the proposed workflow. Each frame in the molecular dynamic (MD) trajectory is represented as a heterogeneous graph, which contains residue and substrate nodes and their residue–residue (RR) and residue–substrate (RS) edges. The model consists of 3 SageConv layers, a pooling layer, a dropout operation, and a linear mapping.

To identify the regioselectivity of GHs, we encoded the structure of the enzyme–substrate complex as a heterogeneous graph and modeled it from MD simulations using energy terms such as electrostatic interactions and van der Waals interactions (Figure 6). We constructed a dataset based on MD trajectories, where each frame of the simulation was abstracted into a heterogeneous graph. The structure and function of the complex system can be more comprehensively described and better understood by integrating information from different data sources in the heterogeneous graph representation. By modeling the enzyme–substrate complexes as heterogeneous graphs, we aim to capture more detailed residue–residue and residue–substrate interactions, facilitating a better understanding and utilization of complex data relationships for more precise and intelligent applications.

We trained our model using 5-fold cross-validation on the training set and evaluated its performance on the glycosyl and replicate test sets (Table 2). In the glycosyl test set, the model achieved a precision of 96.6%, a recall of 100%, an accuracy of 98.1%, and an MCC of 96.4, demonstrating excellent performance in the site classification task of regioselectivity of GHs. To test the generalization ability of the model, our train set and glycan test set are composed of different systems. Our model showed strong generalization ability and accurate identification of the glycan test set. The low variance in these evaluation metrics indicates the high reliability and robustness of our model. Additionally, the uncertainty score of 0.0214 further supports the model’s stability. To illustrate the role of multiple MD trajectory replicates in ML scenarios, we built a replica test set. Our model also performed very well on the replicate test set, suggesting that a model trained on replicates from the same system can accurately recognize other replicate data, implying that multiple MD trajectory replicates are not necessary during the model training process.

### 3.6. Active Learning Weakens Reliance on Molecular Dynamics

**Figure 7:**
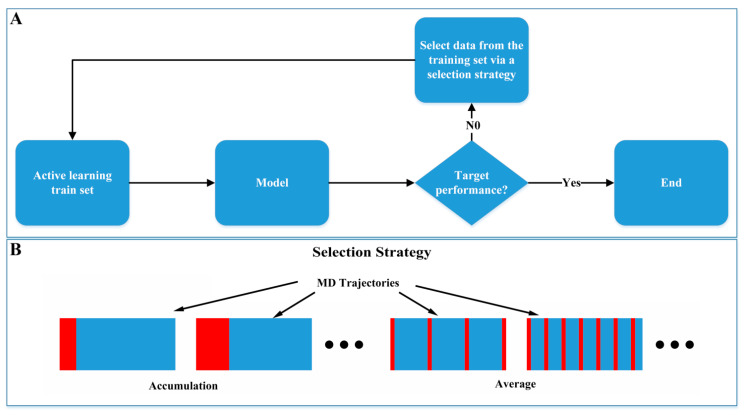
Active learning from molecular dynamics. (**A**) Active learning model training process. (**B**) Active learning training data selection strategy.

To study the impact of simulation duration on model performance, we referred to the idea of the active learning approach, starting with a small amount of training data and gradually increasing the training set to train the model. We selected training data from the training set based on different active learning strategies and evaluated their impact on model performance (Figure 7). Two data selection strategies were employed: (1) Accumulation: Commencing from the start of the MD simulation trajectory and sequentially adding training data. (2) Average: Uniformly selecting the required training data in the whole simulation trajectory.

As shown in Table 3, the performance of models trained with both data selection strategies improved as the amount of training data increased. However, the models trained with the average selection strategy outperformed those trained with the accumulation strategy, revealing that the average strategy captures a broader distribution of data. When the training data increased to 20 ns, the accuracy for the accumulation and average strategies reached 95.2% and 98.1%, respectively, which is very close to the 98.1% accuracy achieved using the full-length MD trajectories, suggesting that extended MD simulations are not necessary. For the task of determining regioselectivity in GHs, about 20 ns simulation data may be sufficient for the training requirements of deep learning models.

### 3.7. Model Interpretability and Its Important Elements

**Figure 8:**
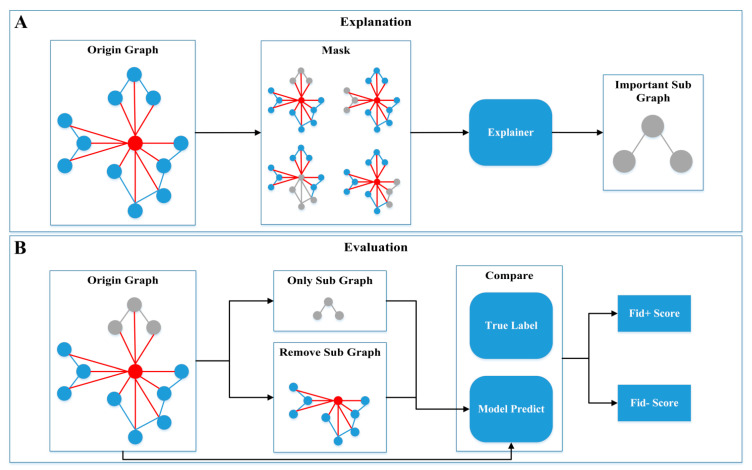
Model interpretability. The explanation (**A**) and evaluation (**B**) process of model interpretability.

Although ML models have achieved high accuracy in modeling MD data, their “black box” nature hinders the understanding of the results and limits their application in biomolecular systems. Therefore, model interpretability algorithms were employed to identify key residues related to regioselectivity in enzyme–substrate interactions. Our approach consists of the following steps (Figure 8): (1) Interactively masking some subgraphs on the original graph and finding the subgraph that has the greatest impact on the model predictions. (2) Integrating the gradients of node features to determine the importance of nodes and features. (3) Evaluating the reliability of the model’s interpretability by comparing fidelity scores before and after removing the important subgraph.

As shown in Table 4, among the five node features with the greatest impact on classification, short-range (elec14) and long-range (elec) electrostatic interaction energy occupies a predominant position, accounting for nearly 100% of the influence. Electrostatic interactions, through the attraction and repulsion between charges, significantly affect the affinity and selectivity between enzymes and substrates, highlighting their crucial role in regioselectivity. We analyzed the top five edges with the greatest impact on classification. In Tpe and Tca, the edges between the enzyme residues and the substrate (RS) are the most critical, accounting for nearly 100% of the influence. In Pfu, both the internal edges between enzyme residues (RR) and the RS edges are important, each contributing approximately 50%. Compared to Pfu, the RS edges in Tpe and Tca are more crucial for determining enzyme regioselectivity. The importance analysis achieved near 1.0 Fid_+_ scores and 0 Fid_-_ scores, indicating that our interpretability is highly reliable. In summary, according to the interpretable algorithm, the most important feature of regioselectivity is electrostatic interaction. In addition, the interpretable algorithm also pointed out that the RS edge is the most important in the edge importance analysis, which means that the electrostatic interaction between the residue and the substrate has the greatest impact on the regioselectivity.

### 3.8. Regioselective Residues Extracted from Model Interpretability

**Figure 9:**
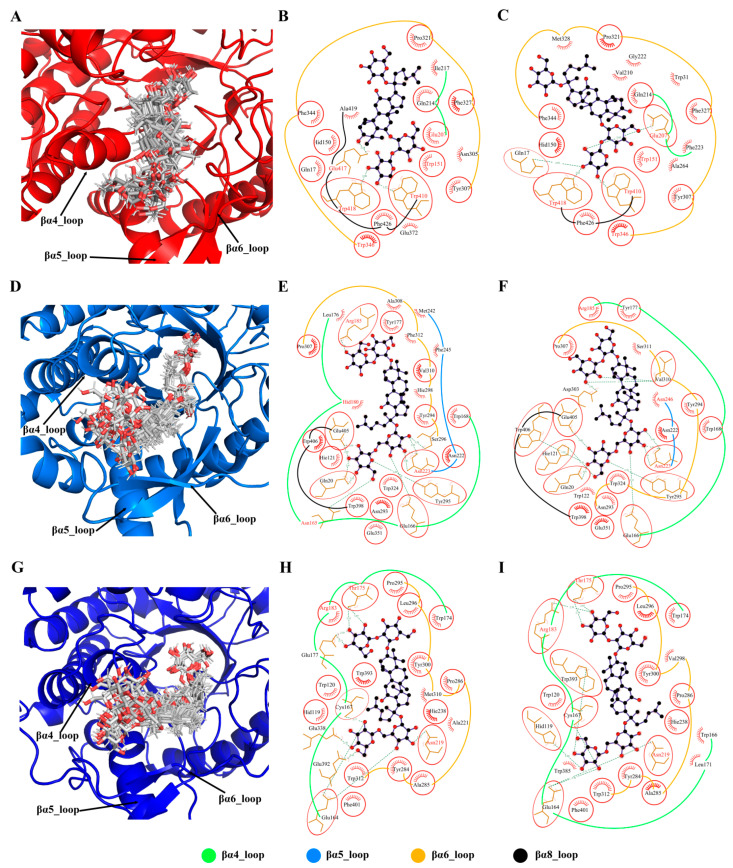
Binding modes of ginsenoside hydrolases with substrates. Conformational clustering of Pfu with substrate R1 (**A**), Tpe with substrate Rb1 (**D**), and Tca with substrate Rb1 (**G**). Binding modes of the representative conformations of Pfu with substrate R1 (**B**,**C**), Tpe with substrate Rb1 (**E**,**F**), and Tca with substrate Rb1 (**H**,**I**). The same residues in the two binding modes are circled in red, and the top 10 important residues are marked in red font.

The top 10 residues most critical for the classification task were identified by model interpretability (Supplementary Figure S4). The cyan boxes in Supplementary Figure S1 mark the top 10 important residues in the sequence. To further determine the residues associated with the regioselectivity of GHs, we clustered the substrate conformations from the MD trajectories and extracted the binding modes of the two main conformations with the largest population (Figure 9).

From the cluster ensemble of substrate R1 conformations in Pfu (Figure 9A), it can be seen that the substrate exhibits a stable binding mode with minimal conformational fluctuations due to the constraint by the enzyme’s catalytic site. However, regions far away from the catalytic site show larger conformational fluctuations. The βα4_loop, βα5_loop, and βα6_loop, being close to the highly fluctuating parts of the substrate, consequently exhibit significant conformational variability. Examining the two primary conformations of substrate R1 in Pfu (Figure 9B,C), the key interacting residues in both binding modes are similar, mainly involving residues on the βα4_loop, βα5_loop, and βα6_loop. Among these interacting residues, six are consistent with model interpretability and five are common to both binding modes Trp151, Glu207, Trp346, Trp410, and Trp418. It should be noted that Glu207 is a catalytic site residue, while hydrophobic Threonine in 151, 346, 410, and 418 positions provide a favorable hydrophobic environment for substrate binding.

Similar to Pfu, the conformational fluctuations of the substrates in Tpe (Figure 9D) and Tca (Figure 9G) were large when they interacted with βα4_loop, βα5_loop, and βα6_loop. In the binding modes of Tpe (Figure 9E,F) and Tca (Figure 9H,I), both of them mainly established strong connections with βα4_loop and βα6_loop. The key residues identified by the model interpretability are mainly distributed in βα4_loop and βα6_loop, implying their strong relationship to the regioselectivity of GHs.

## 4. Conclusions

Our study has significantly advanced the understanding of GHs by identifying regioselective residues using a novel graph-based active learning framework combined with MD simulations. The dynamic-aware graph model demonstrated remarkable accuracy (96–98%) in distinguishing GH regioselectivity, even among enzyme–substrate systems with similar dynamic behaviors. This high level of precision underscores the robustness of our active learning strategy, which efficiently reduces reliance on extensive dynamic data by leveraging short multi-replica simulations. Furthermore, the interpretability of our model allowed us to pinpoint crucial residues and features associated with the regioselectivity of GHs, offering valuable insights into their catalytic mechanisms. These findings not only enhance our fundamental understanding of GHs but also have practical implications. The ability to accurately model enzyme catalytic specificity paves the way for the rational design of GHs, potentially leading to more efficient industrial processes for producing rare deglycosylated bioactive compounds from *Panax* plants. In conclusion, the computational framework we developed bridges the gap between experimental and computational approaches, offering a comprehensive tool for enzyme optimization and design. This integration holds promise for future applications in biotechnology, where tailored enzymatic properties can be engineered to meet specific industrial needs.

## Figures and Tables

**Table 1:** Simulation systems for the regioselectivity of ginsenoside hydrolase.

Regioselectivity	Ginsenoside Hydrolase	Substrate
C-6	*Pfubgl1* (pfu)	R1, R2, Re, Rf, Rg2
C-20	*Tpebgl1* (Tpe)	Rb1, Rb2
	*Tcabgl1* (Tca)	Rb1, Rb2, Rc

**Table 2:** Classification performance of ginsenoside hydrolase regioselective.

Dataset	Precision	Recall	Acc	Mcc	Uncertainty
Glycan test set	96.6 ± 3.8	100.0 ± 0.0	98.1 ± 2.1	96.4 ± 4.0	0.0214 ± 0.0187
Replica test set	98.5 ± 1.4	100.0 ± 0.0	99.2 ± 0.8	98.5 ± 1.5	0.0075 ± 0.0069

**Table 3:** Effect of active learning strategies (accumulation and average) on classification performance of ginsenoside hydrolase regioselective.

Cumulative Data	Accumulation					Average				
	Precision	Recall	Acc	Mcc	Uncertainty	Precision	Recall	Acc	Mcc	Uncertainty
3000 (5 ns)	84.2 ± 8.7	100.0 ± 0.0	90.0 ± 6.4	82.0 ± 10.8	0.0395 ± 0.0033	91.6 ± 6.6	100.0 ± 0.0	95.1 ± 4.0	90.9 ± 7.3	0.0335 ± 0.0180
6000 (10 ns)	86.6 ± 9.2	100.0 ± 0.0	91.6 ± 6.2	84.9 ± 10.8	0.0357 ± 0.0132	92.8 ± 4.0	100.0 ± 0.0	95.9 ± 2.3	92.3 ± 4.3	0.0111 ± 0.0058
12,000 (20 ns)	92.1 ± 8.4	100.0 ± 0.0	95.2 ± 5.4	91.2 ± 9.7	0.0233 ± 0.0104	96.6 ± 4.2	100.0 ± 0.0	98.1 ± 2.3	96.4 ± 4.4	0.0165 ± 0.0160

**Table 4:** The important features and edges identified by the model with validation by Fidelity score.

Ginsenoside Hydrolases	Feature Importance							Edge Importance		Fidelity Score	
	Bond	Angle	Dih	Vdw14	Elec14	Vdw	Elec	RR Edge	RS Edge	Fid_+_ Score (↑)	Fid_−_ Score (↓)
Pfu	0%	0%	0%	0%	96%	0%	4%	54%	46%	0.95	0.05
Tpe	0%	1%	0%	0%	69%	0%	30%	0%	100%	1.00	0
Tca	0%	0%	0%	0%	62%	0%	38%	1%	99%	0.99	0.01

## Data Availability

The data, source code, and trained models are available at https://github.com/liyigerry/DyGNN, (accessed on 19 June 2024).

## Supplementary Materials

The following supporting information can be downloaded at: https://www.mdpi.com/article/10.3390/molecules29153614/s1, Figure S1: Sequence comparison of Pfu, Tpe, and Tca., Figure S2: Structural representation of the substrate, Figure S3: Final docking poses compared to reference structures, Figure S4: Important residues associated with the regioselectivity of ginsenoside hydrolases; Table S1: Details of the final docking result.
