# Supplementary material for: Identify Regioselective Residues of Ginsenoside Hydrolases by Graph-Based Active Learning from Molecular Dynamics
Source: Molecules. 2024 Jul 31;29(15):3614. doi: 10.3390/molecules29153614 (PMC11314057; doi:10.3390/molecules29153614)
Supplement: Supplementary file 1 [file molecules-29-03614-s001.zip › molecules-3092079-supplementary.pdf]

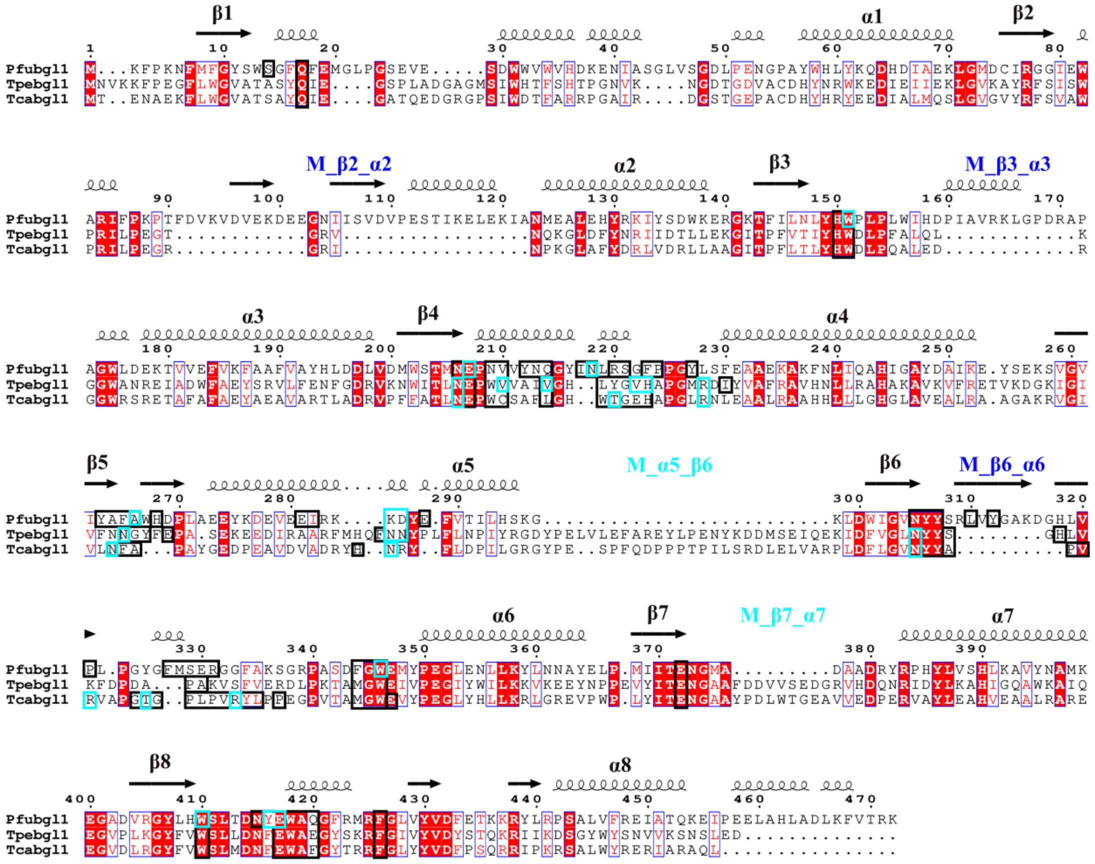

Supplementary Fig. S1. Sequence comparison of Pfu, Tpe, and Tca. Identical or similar residues are framed by blue lines, with identical residues shown as white on a red background and similar residues as red on a white background. The regular secondary structural elements are sequentially numbered and depicted as spirals for α helices and arrows for β strands. Sequence gaps are marked with M. Residues near the substrate on the catalytic surface selected by the graph nodes are highlighted with black boxes. Key residues identified by the model interpretability algorithm are highlighted with cyan boxes.

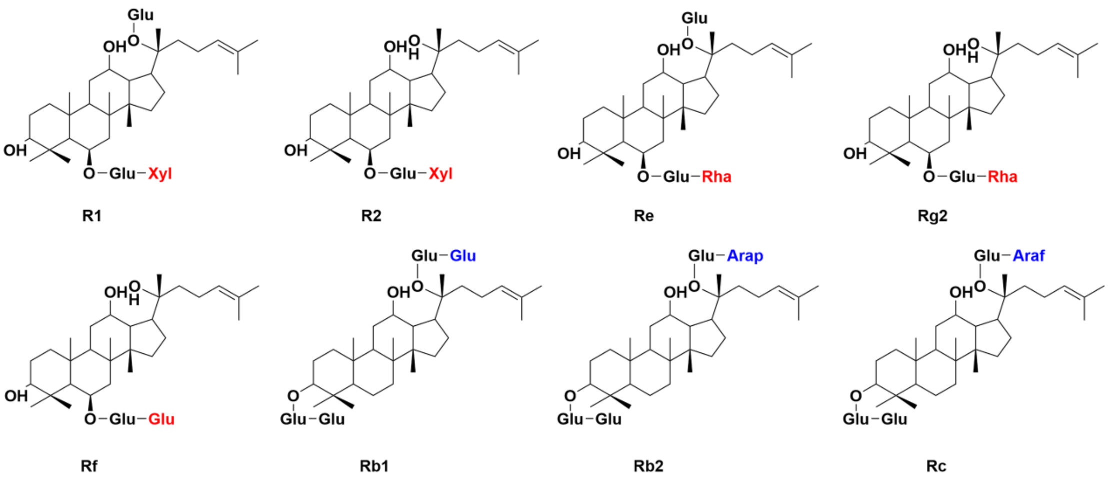

Supplementary Fig. S2. Structural representation of the substrate. The glycosyl groups at positions C6 and C20 are marked in red and blue, respectively.

Supplementary Table S1.

Enzyme	Reference	Substrate	Energy(kcal/mol)	Glycosidic bonds distance(Å)
Pfu	8B81	R1	-8.4	0.65
	2O9R	R2	-9.1	3.08
	2O9R	Re	-8.9	2.36
	2O9R	Rf	-8.4	2.48
	2O9R	Rg2	-9.7	3.53
Tpe	2O9R	Rb1	-7.8	0.82
	2O9R	Rb2	-8	0.75
Tca	2O9R	Rb1	-8.7	0.97
	2O9R	Rb2	-8.3	1.07
	2O9R	Rc	-9.1	0.43

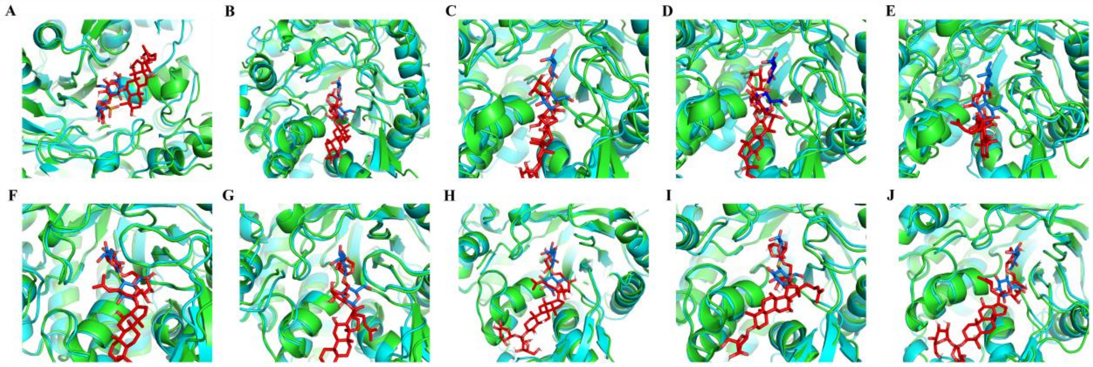

Supplementary Fig. S3. Final docking poses compared to reference structures. A, B, C, D, and E: Final docking results of Pfu with R1, R2, Re, Rg2, and Rf, respectively. F and G: Final docking results of Tpe with Rb1 and Rb2, respectively. H, I, and J: Final docking results of Tca with Rb1, Rb2, and Rc, respectively. The substrates in ginsenoside hydrolases and reference structures are shown in red and blue, respectively.

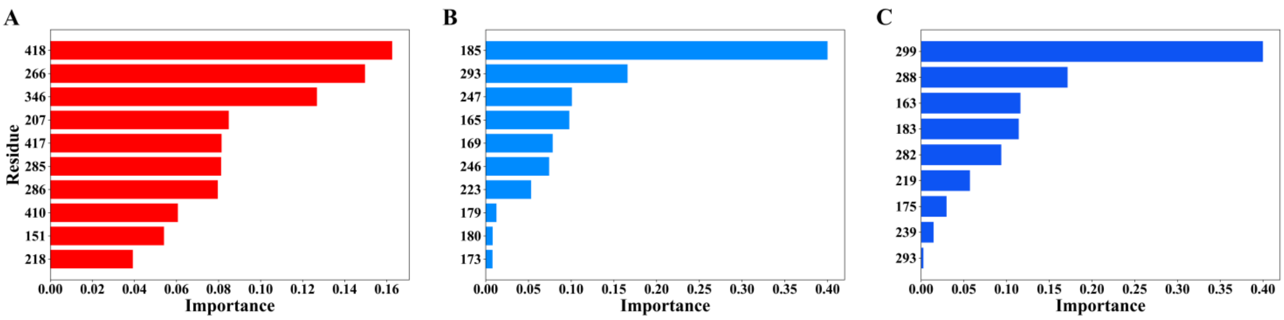

Supplementary Fig. S4. Important residues associated with the regioselectivity of ginsenoside hydrolases. The top 10 important residues of Pfu1 (A), Tpe (B), and Tca (C).
